# Aqueous Pb(II) Removal Using ZIF-60: Adsorption Studies, Response Surface Methodology and Machine Learning Predictions

**DOI:** 10.3390/nano13081402

**Published:** 2023-04-18

**Authors:** Usman M. Ismail, Sagheer A. Onaizi, Muhammad S. Vohra

**Affiliations:** 1 Civil and Environmental Engineering Department, King Fahd University of Petroleum & Minerals (KFUPM), Dhahran 31261, Saudi Arabia; g202101790@kfupm.edu.sa; 2 Chemical Engineering Department, King Fahd University of Petroleum & Minerals (KFUPM), Dhahran 31261, Saudi Arabia; onaizi@kfupm.edu.sa; 3 Interdisciplinary Research Center for Hydrogen and Energy Storage, King Fahd University of Petroleum & Minerals (KFUPM), Dhahran 31261, Saudi Arabia; 4 Interdisciplinary Research Center for Construction and Building Materials, King Fahd University of Petroleum & Minerals (KFUPM), Dhahran 31261, Saudi Arabia

**Keywords:** lead removal, adsorption isotherm and kinetics, response surface methodology (RSM), machine learning, water treatment, heavy metals

## Abstract

Zeolitic imidazolate frameworks (ZIFs) are increasingly gaining attention in many application fields due to their outstanding porosity and thermal stability, among other exceptional characteristics. However, in the domain of water purification via adsorption, scientists have mainly focused on ZIF-8 and, to a lesser extent, ZIF-67. The performance of other ZIFs as water decontaminants is yet to be explored. Hence, this study applied ZIF-60 for the removal of lead from aqueous solutions; this is the first time ZIF-60 has been used in any water treatment adsorption study. The synthesized ZIF-60 was subjected to characterization using FTIR, XRD and TGA. A multivariate approach was used to investigate the effect of adsorption parameters on lead removal and the findings revealed that ZIF-60 dose and lead concentration are the most significant factors affecting the response (i.e., lead removal efficiency). Further, response surface methodology-based regression models were generated. To further explore the adsorption performance of ZIF-60 in removing lead from contaminated water samples, adsorption kinetics, isotherm and thermodynamic investigations were conducted. The findings revealed that the obtained data were well-fitted by the Avrami and pseudo-first-order kinetic models, suggesting that the process is complex. The maximum adsorption capacity (*q*_max_) was predicted to be 1905 mg/g. Thermodynamic studies revealed an endothermic and spontaneous adsorption process. Finally, the experimental data were aggregated and used for machine learning predictions using several algorithms. The model generated by the random forest algorithm proved to be the most effective on the basis of its significant correlation coefficient and minimal root mean square error (RMSE).

## 1. Introduction

Demand for water is increasing across the globe because of industrial and agricultural growth, and the steady increase in the human population. Additionally, the rate at which water bodies are contaminated endangers human health [1,2]. Thus, the World Health Organization (WHO) projects that by 2025, about 50% of the global population will suffer from freshwater shortage [3]. Thus, it is essential to treat water as a valuable resource. Heavy metals as a class of inorganic contaminants are considered both toxic and recalcitrant pollutants present in polluted waters [4]. Because of their widespread use in industrial processes, they are injected into aquatic streams as part of industrial effluent. Many of the heavy metals are hazardous substances that are mutagenic, carcinogenic and teratogenic that can migrate with water flow, as such presenting multiple routes for human exposure. Further, they can be taken up by plants and animals resulting in bioaccumulation and biomagnification [5,6,7]. In particular, ingestion of lead above the permissible limit (10 and 15 ng/L according to WHO and USEPA, respectively), can result in hepatitis, anemia, encephalopathy and nephritic syndrome [8]. Thus, it is important to find innovative ways for the effective removal of lead from aqueous systems.

Many technologies including electrolysis [9], ion exchange [10], chemical precipitation [11], coagulation [11,12], phytoremediation [13] and adsorption [14] have been applied and improved for the treatment of water contaminated with lead [15]. While every method has its own advantages, most of them suffer from certain drawbacks that limit their applicability to industrial-scale operations. However, adsorption has emerged as a suitable treatment method due to its simplicity, efficiency, low cost and ability to remove multiple pollutants from waste streams [16,17]. Traditional adsorbents such as activated carbon, chitosan, biomass, molecular sieves and polymers have been widely used to remove water pollutants. However, their adsorption capacity is often not high, in addition to their poor selectivity towards many contaminants [18], making the development of more effective adsorbents a necessity.

Zeolitic imidazolate frameworks (ZIFs), a subclass of the metal–organic frameworks (MOFs), have recently emerged as attractive materials for various applications such as drug delivery, catalysis and energy storage [19,20,21]. ZIFs are crystalline materials with large porosity and specific surface area which may reach up to 10,000 m^2^/g, making them interesting candidates for the adsorption of contaminants from water [20,22]. They also usually possess better thermal and chemical stabilities relative to MOFs. ZIFs’ permanent porosity allows for the easy introduction of functional groups which can help increase active sites for efficient adsorption, hence, creating functional materials [22]. Further, the structure of ZIFs which is made up of a metal node, organic linker and available surface functional groups leads to its several documented heavy metal removal mechanisms including ion exchange, pore diffusion, coordination and many others makes them appealing adsorbents [23,24]. However, so far, studies on the application of ZIFs for water decontamination mainly focus on ZIF-8 and ZIF-67. Ahmad et al. [25] synthesized both ZIF-67 and ZIF-8 and applied them for the removal of lead from contaminated water with adsorption capacity reaching up to 1978.6 and 1780.9 mg/g, respectively. Huang et al. [26] also developed ZIF-67 and ZIF-8 and tested their performance for lead adsorption from aqueous solutions; they found that the lead adsorption capacity reached 1348.4 and 1119.8 mg/g, respectively. Although ZIF-8 and ZIF-67, and their composites, have been widely studied in the literature, the application of other ZIF materials (e.g., ZIF-60) for water decontamination is still greatly lacking.

Therefore, the key objective of this study is to investigate the removal of lead from contaminated water samples using ZIF-60. To the best of our knowledge, this is the first study on utilizing ZIF-60 for the adsorptive removal of any water pollutants. The effect of varying the process parameters on the removal of lead was studied using the response surface methodology (RSM) technique and regression models were developed using the same technique. Furthermore, classical adsorption studies including adsorption kinetics, isotherms and thermodynamics were also conducted. Finally, the obtained experimental data were used to develop machine learning algorithms that were used for the prediction of lead removal from aqueous solutions using ZIF-60 adsorbent. The field of machine learning and artificial intelligence is gaining prominence in many disciplines; however, it has been scarcely applied in aspects pertaining to the use of novel materials for water treatment applications. Hence, another key objective of this study is to use the obtained experimental data for testing the predictive performance of machine learning algorithms in terms of lead removal from contaminated water samples using ZIF-60 material.

## 2. Materials and Methods

### 2.1. Chemicals and Analytical Instruments

The reagents employed in this study were of high purity including zinc nitrate hexahydrate (Sigma-Aldrich, USA), imidazole (Sigma-Aldrich, USA), 2-methylimidazol (Sigma-Aldrich, USA), N,N-dimethylformamide (Honeywell, USA), tri-ethyl amine (Sigma-Aldrich, USA), sodium hydroxide (EMSURE^®,^ USA), lead nitrate (BDH, UK), hydrochloric acid (Fisher Scientific, USA). Ultra-pure water (CORNING Mega Pure^TM^, USA), was used for sample preparation and dilution. The glassware used was thoroughly washed, cleaned and dried before each use.

X-ray diffractometer (XRD Rigaku Miniflex-II, Japan) was used to investigate the crystal phases of the synthesized ZIF-60; current was set at 15 mA while the CuKα1 radiation was kept at 40 kV. The 2θ angle range for the analysis was set between 5 to 40° at a scanning rate of 7.00°/min. Thermal stability of ZIF-60 was tested using thermogravimetric analysis (TA Instruments) in N_2_ atmosphere using a heating rate of 10 °C/min in the temperature range from 30 to 800 °C. FTIR spectra were collected using Smart iTR NICOLET iS10 FTIR spectroscopy for surface functional groups investigation.

The lead concentration in the aqueous solutions was determined using atomic absorption spectroscopy (PerkinElmer, USA); the samples were passed through 0.2 µm filter (Whatmann, Germany) before subjecting them to analysis. pH measurements were conducted using OAKTON ^®^ pH 700.

### 2.2. Synthesis of ZIF-60

Herein, the solvothermal method was employed for the synthesis of ZIF-60 as reported by Yaghi group [27] with some modifications. Briefly, 2.68 g zinc nitrate hexahydrate was added to 60 mL N,N-dimethylformamide (DMF, USA) and stirred till it dissolved. A separate solution was prepared by adding 1.84 g imidazole and 0.74 g 2-methylimidazol and stirred till completely dissolved. The two solutions were then mixed together, and 1 mL tri-ethyl amine was injected into the mixture. The obtained solution was then stirred for 1 h, at room temperature, with the aid of a magnetic stirrer while the beaker was carefully covered with aluminum foil to avoid the absorption of water moisture. The mixture was then transferred to an autoclave reactor and incubated in an oven (Fisher Scientific, USA) for 48 h at 85 °C. Afterward, synthesized ZIF-60 was first washed with N,N-dimethylformamide using a centrifuge (Sigma Laborzentrifugen GmbH, Germany) followed by several washes with de-ionized water. The washed sample was then placed in the oven at 50 °C until it completely dried out. The dried ZIF-60 was stored in tightly closed vials until use.

### 2.3. Experimental Design

The study made use of a multivariate approach instead of the traditional univariate approach to conducting the experiments. The effects of the process parameters and their interaction were explored using the RSM technique. The experiments were designed with three factors in a typical face-centered central composite design [28]. The independent variables of the study include ZIF-60 dose, lead initial concentration, and the temperature at which the experiments were conducted while the dependent variable (response) was the percentage removal of lead. The factors of interest and their respective levels are presented in Table 1.

Several regression models were generated for response prediction, and the model that provided the best fit of the experimental data was selected and reported based on several quality parameters including the coefficient of determination (R^2^), adjusted and predicted R^2^. Along the same line, the analysis of variance (ANOVA) was used to confirm the validity and performance of the generated models. Design Expert version 13 software was employed for the RSM modeling and ANOVA technique.

### 2.4. Batch Adsorption Experiments

Initially, a 1000-ppm stock solution of lead was prepared by dissolving the analytical grade lead in de-ionized water. Serial dilution was then used to prepare the desired lead concentrations. For each test, 100 mL of the desired lead concentration was placed in 125 mL Pyrex flask prior to the addition of the required adsorbent dose. The mixture was then placed inside an incubator shaker (Bioevopeak, USA) and kept for 24 h at the required temperature. An agitation speed of 250 rpm was used throughout the experiments. The lead removal efficiency and the adsorption (uptake) capacity were then computed using Equations (1) and (2), respectively.

(1) $$R=\frac{Co-Ce}{Co}\times 100$$

(2) $$qe=\frac{Co-Ce}{m}\times V$$

where *R*, *qe*, *C_o_* and *C_e_* represent the removal efficiency (%), adsorption capacity (mg/g), lead initial concentration (ppm) and lead concentration (ppm) after 24 h of experiment. *V* represents sample volume in liters and m is the amount of adsorbent added in g.

For adsorption kinetics experiments, a ZIF-60 dose of 0.025 g/L was used while the lead initial concentration was fixed at 150 ppm. The temperature was kept at 25 °C and aliquots were collected at different time intervals between 10 and 1440 min and subjected to analysis. Adsorption isotherm experiments were conducted for 24 h at 25 °C, a ZIF-60 dose of 0.025 g/L was added while the lead initial concentration was varied over a range of 10–400 ppm. As for thermodynamics study, a ZIF-60 dose of 0.05 g/L was fixed as well as an initial lead concentration of 100 ppm using an equilibration time of 24 h. The temperature was varied within the range of 25–45 °C. All lead solutions were prepared at an initial pH of 4 to ensure that the removal is attributed to the addition of ZIF-60 and not lead hydroxide precipitation. Lead exists mainly as Pb(II) at pH less than 6.0; however, slightly above this pH, precipitates and other Pb compounds begin to appear [29,30].

### 2.5. Machine Learning Algorithms

The use of machine learning algorithms for the prediction of adsorbent performance in removing contaminants from aqueous streams is gaining prominence [31,32,33]. However, the application of machine learning and other artificial intelligence approaches to the field of wastewater treatment is limited by the availability of experimental data due to various cost and operational factors. As such, this study collated the batch adsorption experimental data that were obtained and used them to test the predictive performance of several machine learning algorithms. The statistical summary of the dataset used for this investigation is given in Table 2. Further, short descriptions of the working principles of the algorithms used in this study are also presented below.

#### 2.5.1. Artificial Neural Network (ANN)

**Figure 1:**
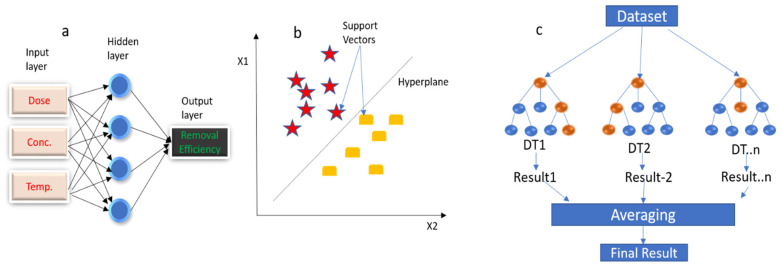
(**a**) Structure of the ANN algorithm, (**b**) SVM classification using linear hyperplane, (**c**) architecture of the RF algorithm.

ANN is also termed a multi-layer perceptron and it is one of the most widely used artificial intelligence techniques. It drives its concept from the mathematical delineation of the nervous system [34]. The structure of ANN consists of three distinct layers with the first recognized as the input layer while the last is the output layer. In between them is the hidden layer which can be one or several. The layers consist of multiple neurons in the form of nodes that are connected to each other. Weights between the nodes can be altered to enhance the model performance with the help of an appropriate cost function. Activation functions such as sigmoid and rectified linear units (ReLU) are used for nonlinear computations. Backpropagation technique is often used to train the model [35]. The illustration of the feed forward-ANN is given in Figure 1a, and the ANN output prediction is given by Equation (3).

(3) $$y=f\left(W+B\right)$$

y represents the output, W is the weight, B stands for bias and f is the activation function.

#### 2.5.2. Support Vector Machine (SVM)

While the SVM was originally devised to solve classification problems, it has been developed to solve regression problems even with relatively small-sized datasets [36]. This is because the SVM works on the concept of mapping input vectors into feature space that is of high dimension. This enables the SVM to distinguish between two classes in the feature space by drawing a hyperplane between the increased margin separating the classes involved [37]. Different kernel functions are used to obtain the mapping. The working principle of the SVM is depicted in Figure 1b while Equation (4) is used to generate the output.

(4) $$y=\overset{n}{\underset{i=1}{\sum }}\alpha iK\left(xi,xj\right)+b$$

where *y* is the predicted output value, *αi* is the Lagrenge multiplier, *K(xi,xj)* is the kernel function and *b* represents a threshold parameter.

#### 2.5.3. Random Forest (RF)

The random forest is considered an ensemble learning technique that uses several decision trees to solve regression or classification problems. The random forest was derived to solve the inherent problems including computational cost and overfitting that are associated with the decision tree (DT). Output prediction with the random forest begins with a random sampling with replacement. Several decision trees are generated and run independently with their outputs finally aggregated to come up with a single output [38]. The schematic representation of the random forest working principle is given in Figure 1c.

The Waikato Environment for Knowledge and Analysis (WEKA) version 3.8.6 was used to build the above prediction models. The N-fold cross-validation was used for training and validation of the generated models. To evaluate model performance, correlation coefficient (R), R^2^ and RMSE were used.

## 3. Results and Discussion

### 3.1. Material Characterizations

**Figure 2:**
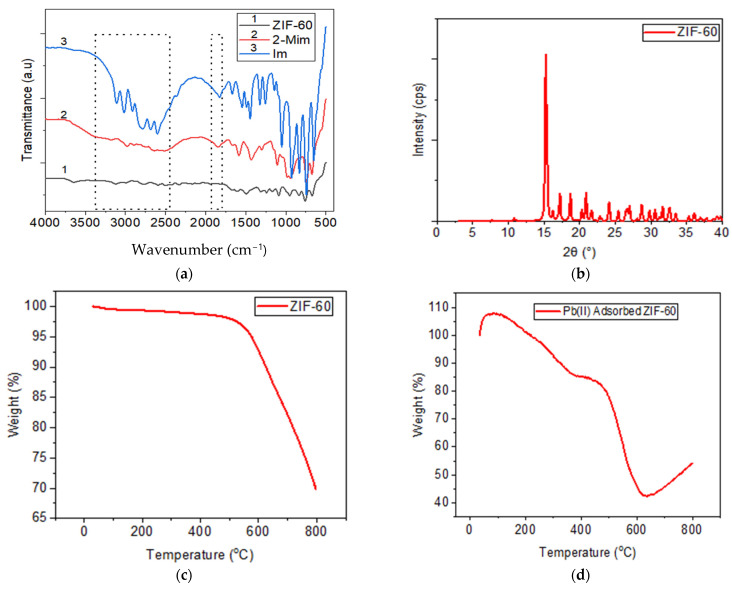
(**a**) FTIR spectra of ZIF-60 and organic precursors, (**b**) XRD pattern of ZIF-60, (**c**) TGA analysis plot of ZIF-60 and (**d**) TGA analysis plot of post lead adsorbed ZIF-60.

To confirm the formation of ZIF-60, a comparison between the FTIR spectra of the organic linkers (imidazole and 2-methylimidazole) and the as-synthesized ZIF-60 was conducted. As shown in Figure 2a, the various broad and strong N-H bands between around 3300 and 2500 cm^−1^ present in the FTIR spectra of the precursors have disappeared from the spectra of the as-synthesized ZIF-60. Further, the sharp peak at around 3125 cm^−1^ and the weak band close to 1815 cm^−1^ ascribed to C-H vibration are also not present in the as-synthesized ZIF-60 FTIR spectra. This gives an indication that the imidazole and 2-methylimidazole are fully deprotonated upon the formation of ZIF-60 structure [39,40].

In addition to FTIR analysis, XRD characterization of the as-synthesized ZIF-60 was also conducted, and the results are shown in Figure 2b. The intense peaks at several positions of the XRD profile of ZIF-60 displayed indicate the crystalline structure of the synthesized material. Peaks appeared at diffraction angles of 7.68, 10.80, 15.24, 16.20, 17.25, 18.69, 27.00, 31.65 and 36.03° are similar to those found in other ZIF structures [38,39]. The most intense peak was noted at a diffraction angle of 15.24°, and it is in an agreement with the previous literature in which ZIF-60 was synthesized [27,39].

To probe the thermal stability of the as-synthesized ZIF-60, the material was subjected to thermal treatment under an inert atmosphere (i.e., nitrogen) up to 800 °C, and results are shown in Figure 2c. As displayed in this figure, there is a negligible material loss (less than 2.5 wt.%) upon heating ZIF-60 up to 520 °C. This signifies that the as-synthesized ZIF-60 is as thermally stable as other ZIF materials [27,41]. Increasing the temperature beyond 520 °C resulted in an accelerated decrease in the sample weight. This relatively fast weight loss is due to thermal decomposition of the ZIF structure [42,43]. Despite the significant material loss from the ZIF-60 framework at high temperatures (i.e., >520 °C), which is also the case for other ZIFs [27,42,43,44,45,46], this is not a concern for utilizing ZIF-60 for mild to moderate temperature processes. TGA analysis was conducted post-lead adsorption (Figure 2d) and the lead adsorbed ZIF-60 showed somewhat less stability at higher temperatures that could result from respective adsorption interaction.

### 3.2. Response Surface Methodology and Analysis of Variance

The adsorption experiments were conducted based on the runs generated by the RSM model and the full design matrix together with the experimental lead removal (%) presented in Table 3. It is noted that at least 99.0% lead removal is achieved when an adsorbent dose of 0.075 g/L is used and lead initial concentration is kept at 50 ppm, independent of the adsorption temperature. Overall, the experiments indicate that ZIF-60 can be used to efficiently remove lead from contaminated water streams based on the excellent performance of ZIF-60 at varied initial concentrations of lead as shown in Table 3.

**Figure 3:**
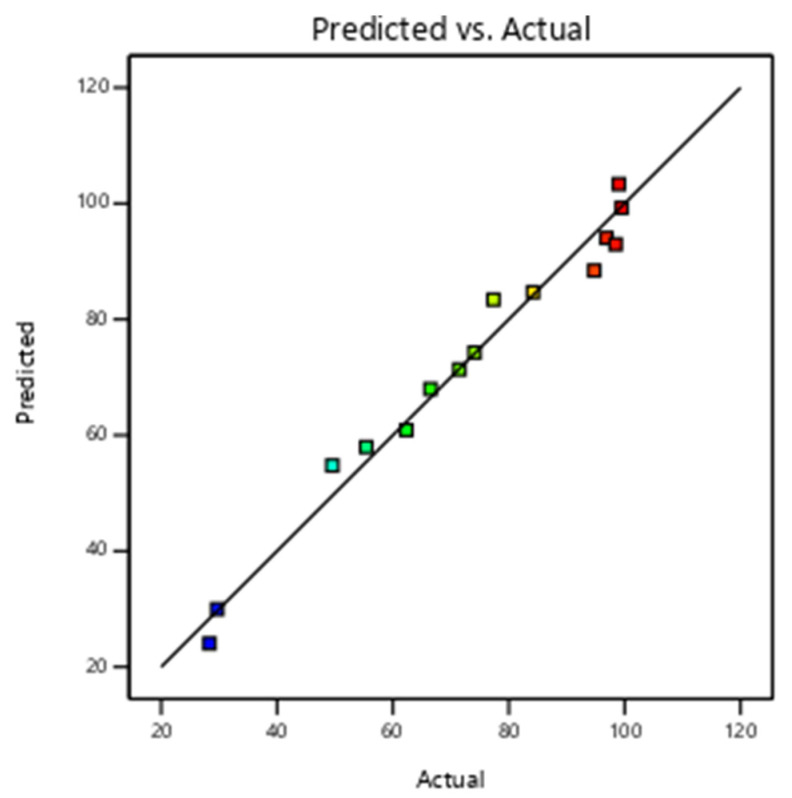
Plot of predicted lead removal efficiency vs. actual lead removal efficiency.

The results were used to generate regression models, and the quadratic model was selected because it provided a strong correlation between the experimental and the predicted lead removal. The R^2^, adjusted R^2^ and predicted R^2^ values were found to be 97.65, 93.41 and 78.22%, respectively. The visualization of the good fitting between the actual and the predicted lead removals is given in Figure 3. The difference between the adjusted and predicted R^2^ was found to be less than 20%, which further signifies the adequacy of the quadratic model [47,48]. The generated quadratic model is given by Equation (5).

Removal Efficiency = 84.70 + 18.05 A − 19.09 B + 2.50 C + 2.24 AB +1.87 AC + 2.33 BC − 8.70 A^2^ − 10.83 B^2^ + 1.23 C^2^ (5)

The RSM-based results were further subjected to analysis of variance to understand the factors that most significantly affect the response and also to reveal the effect of varying the process parameters on the removal of lead from the aqueous system under study. The ANOVA analysis results are shown in Table 4. The significance of the model and its terms were studied using a confidence interval of 95%. The *p*-value of the model is less than 5%, indicating its significance [49,50]. Additionally, the ZIF-60 dose, lead concentration and the quadratic effect of the initial lead concentration were found to be the factors that most significantly influence the output of the generated model.

**Figure 4:**
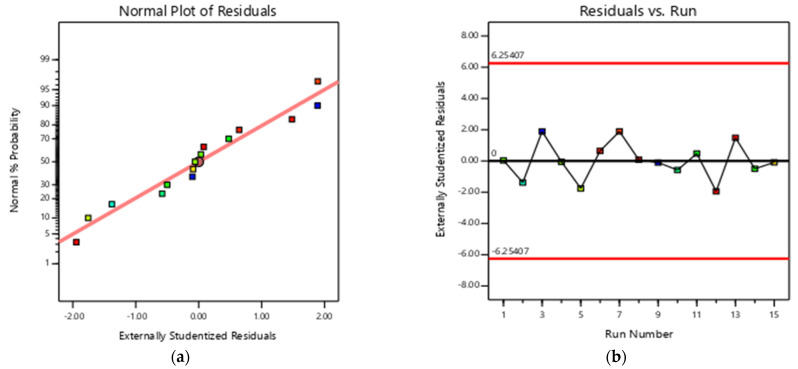
Model adequacy check plots (**a**) normal probability plot (**b**) plot of residuals vs. order of run.

To further validate the generated model, it was tested to ensure that it did not violate the assumptions of the error term. Figure 4a proved that the residuals are normally distributed, while Figure 4b shows that there was no dependence between the order of run and the residuals that were generated; this is signified by the random pattern exhibited by the points in the plot.

**Figure 5:**
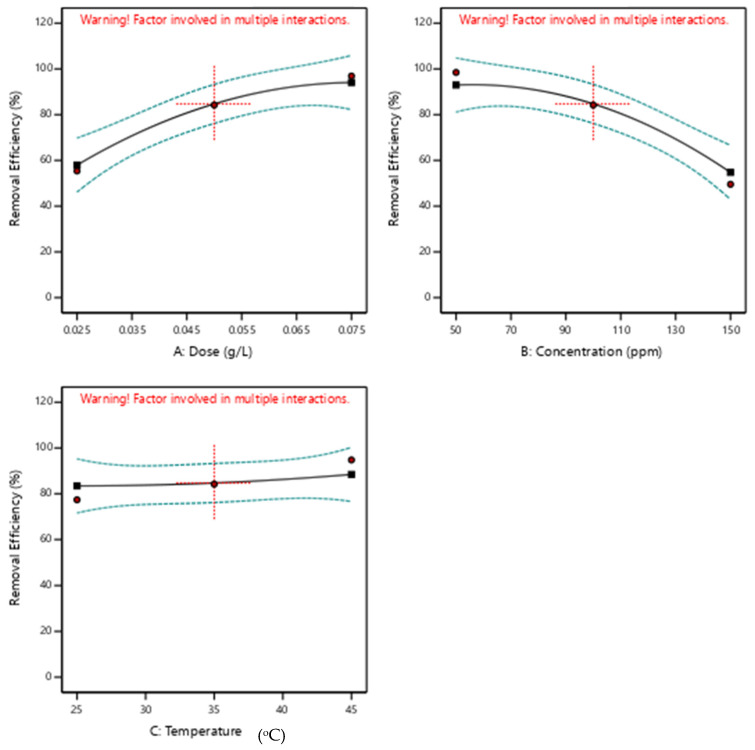
Effect of (**A**) adsorbent dose (**B**) initial lead concentration (**C**) temperature on lead removal efficiency.

The effect of the respective process parameters on lead removal was also investigated based on the ANOVA results. The factorial plots in Figure 5 show how varying the independent variables causes a reduction or increment in lead removal. Figure 5A shows that an increase in ZIF-60 dose leads to an improvement in the removal efficiency; this can be attributed to the increase in the number of available adsorption sites for the uptake of lead from the aqueous stream [40,51]. In contrast, Figure 5B indicates that an increase in the initial lead concentration is detrimental to its removal. This decrease in lead removal with increasing its initial concentration is due to the minute adsorbent dose used in the experiments. At low adsorbent dosages such as the ones used in this study, the adsorption sites get saturated, and the remaining lead cannot be taken up by the saturated ZIF-60 [40,51]. However, if the adsorption capacity is considered, then an increase in initial concentration is beneficial to the uptake of lead because the mass transfer driving force for lead to accumulate at the adsorbent surface increases in this case [52].

Based on the RSM design space, the effect of the temperature was found not to be significant as shown in Figure 5C. However, the trend in the plot indicates that an increase in the temperature favors the adsorption of lead by the ZIF-60, hence, suggesting that the process is endothermic in nature. Further exploration of the effect of the temperature is reported in the adsorption thermodynamics investigation section presented and discussed later. The outcome of the RSM study guided us in selecting the values of the adsorption parameters that were used in the subsequent adsorption studies. It was kept in mind that the temperature was not significant, whereas a high adsorbent dose and low lead concentration would likely favor the uptake of Pb(II) by ZIF-60.

**Figure 6:**
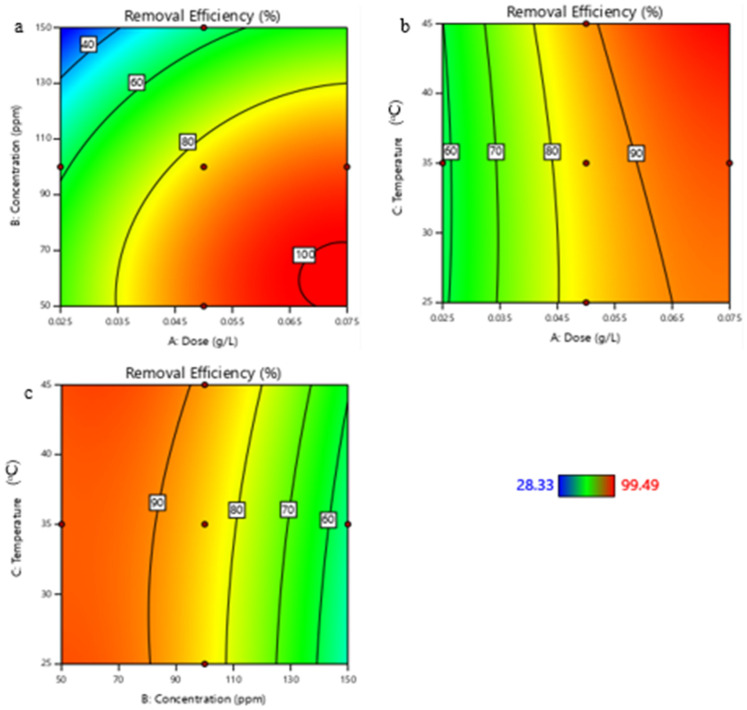
Contour plots for the interaction between (**a**) adsorbent dose and initial lead concentration (**b**) adsorbent dose and temperature (**c**) initial concentration and temperature.

One major advantage of using the multivariate approach over the one factor-at-a-time approach is the ability to study the effect of the interaction between the independent variables on the response of interest (lead removal in this study). The contour plots showing the effect of the interaction are given in Figure 6a–c. From Figure 6a, it is noted that a near complete removal is noted when the adsorbent dose of 0.075 g/L is used, and the initial lead concentration is kept at 50 mg/L. The contour plots in Figure 6a–c also support the finding that the initial lead concentration and ZIF-60 dose are the most important factors affecting the lead removal efficiency based on the RSM design that was adopted in the study.

### 3.3. Adsorption Kinetics

**Figure 7:**
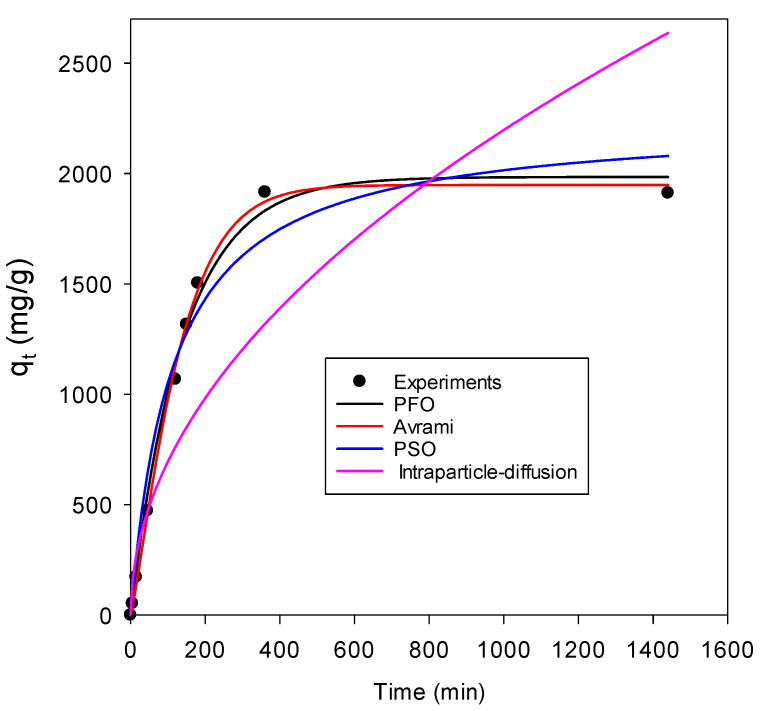
Fitting of kinetics models to experimental data for lead adsorption onto ZIF-60 (*C_o_* = 150 ppm, initial pH = 4, ZIF-60 dose = 0.025 g/L, temperature = 298 K).

Adsorption kinetics equations play a vital role in elucidating adsorption pathways and in providing room for hypothesizing adsorption mechanisms for a specific pollutant under certain adsorption conditions. Information regarding how the adsorption rate is affected by the amount of sorbent present in the contaminated water sample or the adsorption capacity of the material under study is obtained after conducting kinetic studies [53]. As such, the adsorption kinetics data for the uptake of lead using the ZIF-60 material was obtained and fitted to kinetic models including the pseudo-first-order, Avrami and intraparticle diffusion models. The plot of the experimental data fitted to the respective models is presented in Figure 7.

As evident in Figure 7, the pseudo-first-order (PFO) kinetic model, the Avrami model and to a lesser extent, the pseudo-second-order (PSO) kinetic model all reasonably fit well the experimental data, indicating that the uptake of lead by ZIF-60 is a complex process and both physisorption and chemisorption processes are involved in controlling the adsorption rate [22,54]. However, caution needs to be taken when using some of these kinetic models to elucidate the reaction mechanism as they are empirical in nature [55]. The constants generated for the utilized kinetic models are given in Table 5. The experimental data showed relatively poor fitting to the intraparticle diffusion model suggesting that the intraparticle diffusion is not the rate-limiting step of lead adsorption onto ZIF-60 [29]. Further, the R^2^ for the fitted experimental data with reference to the kinetic models was obtained and the values are also reported in Table 5.

### 3.4. Adsorption Isotherms

**Figure 8:**
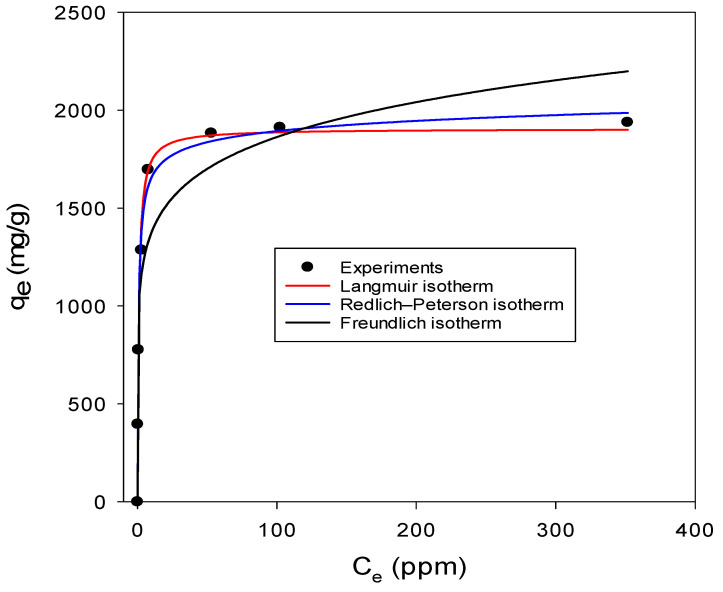
Adsorption isotherm models for lead uptake by ZIF-60 (*C_o_* = 10–400 ppm, initial pH = 4, ZIF-60 dose = 0.025 g/L, temperature = 298 K).

The study of isotherm data is the most adopted approach used for exploring the mechanism of adsorption which is important to the design of adsorption systems and adsorbents. It also gives information about the maximum adsorption capacity (*q*_max_), a significant parameter in determining the suitability of an adsorbent for a certain sorbate [55]. Consequently, an adsorption isotherm study was conducted on the ZIF-60 material to understand its uptake behavior towards lead at the given experimental conditions. The isotherm data obtained were fitted to the Freundlich, Langmuir and Redlich–Peterson (R–P) isotherms as shown in Figure 8. The experimental data fitted well to the Langmuir model as evidenced by the obtained R^2^ value of 0.9817 which is significantly greater than the value obtained when the data were fitted to the Freundlich model. This suggests that the adsorption of lead to ZIF-60 is macroscopic homogeneous adsorption and not multi-layer adsorption on the heterogamous surface [55,56]. The R–P isotherm is a three-parameter model that was developed to overcome the shortcomings of both the Freundlich and Langmuir models [57]. The obtained experimental data are fitted well with the Redlich–Peterson model as shown in Figure 8; this is not surprising because as the value of the constant g approaches 1, the Redlich–Peterson isotherm can be approximated as the Langmuir isotherm [56]. The ZIF-60 material showed an impressive lead uptake capacity with *q*_max_ predicted using the Langmuir isotherm equal to 1905.0 mg/g; this value is very close to the experimental adsorption capacity obtained at the highest lead initial concentration of 400 ppm utilized in this study, which is 1938.0 mg/g. The lead uptake capacity by ZIF-60 obtained in this study is significantly higher than what was obtained by many other adsorbents (to be presented later), which signifies that the ZIF-60 material can be utilized for the removal of lead from aqueous systems. The parameters of the respective isotherm models are given in Table 6.

### 3.5. Thermodynamic Studies

**Figure 9:**
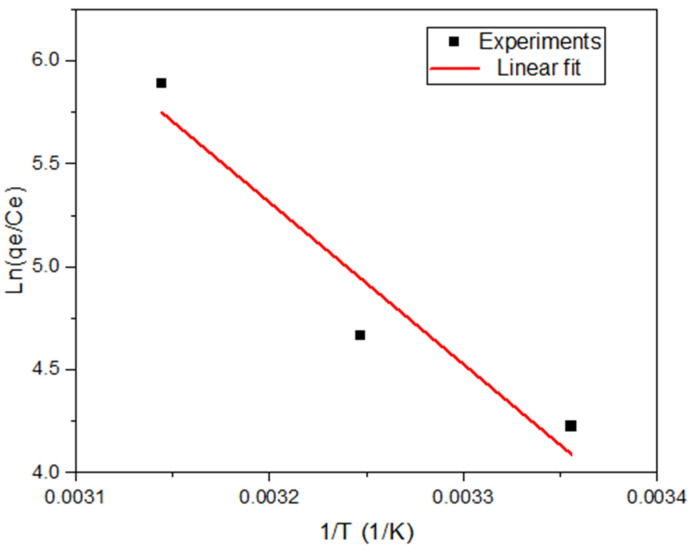
Van’t Hoff linear plot for effect of temperature on lead adsorption onto ZIF-60 (*C_o_* = 100 ppm, ZIF-60 dose = 0.05 g/L, initial pH = 4).

The adsorption temperature influences the equilibrium adsorption capacity as well as the rate at which solute diffuses through the external boundary layer and into the internal pores of the adsorbent [58]. Knowledge about the thermodynamic parameters of an adsorption process helps in determining a suitable temperature for conducting the adsorption process and also gives an insight into the mechanism through which the process occurs. As such, a thermodynamic analysis of the adsorption of lead onto ZIF-60 was conducted and the parameters of interest including the enthalpy change (ΔH), entropy change (ΔS) and standard free energy change (ΔG) are reported in Table 7. The parameters were computed using the van’t Hoff model [8,29] and the linear plot is shown in Figure 9.

The negative values of ΔG indicate that the adsorption process is feasible and spontaneous under the given experimental conditions. Furthermore, an increase in temperature resulted in a more negative ΔG suggesting that increasing the temperature results in more spontaneity. The analysis also revealed a positive ΔH indicating that the adsorption of lead onto ZIF-60 is a heat-absorbing process and increase in temperature favors the adsorption capacity (i.e., an endothermic adsorption process) [41]. This finding supports the hypothesis that was made earlier while analyzing the RSM results that the adsorption process is endothermic in nature. The positive ΔS indicates significant randomness in the solid-solution layer of the adsorption system and that the adsorption of lead onto ZIF-60 is an entropy-driven process [41,59].

### 3.6. Performance of Machine Learning Algorithms in Predicting ZIF-60 Lead Removal Efficiency

Prior to model development, a hyperparameter tuning exercise was conducted. This was completed to ensure that the optimum conditions to maximize the performance of each of the tested algorithms are ascertained. The ANN, SVM and RF have their own respective hyperparameters, and the hyperparameters along with the values that they were set at are reported in Table 8.

**Figure 10:**
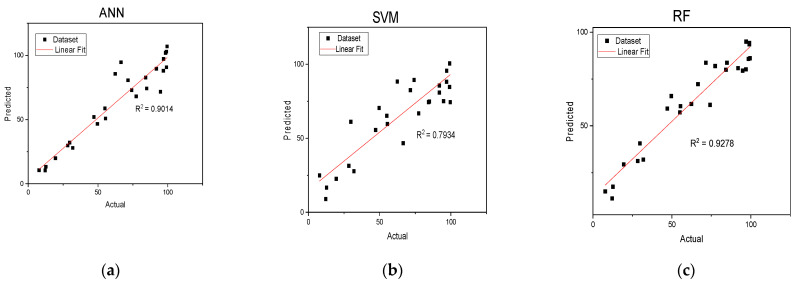
Plots of actual vs. predicted lead removal efficiencies using (**a**) ANN (**b**) SVM (**c**) RF models.

All the optimized models provided a reasonably high correlation between the actual and predicted values. The Pearson correlation coefficient ^®^ was found to be 0.9632, 0.9494 and 0.8907 for RF, ANN and SVM, respectively. Similarly, the root mean squared error (RMSE) was found to be 9.13, 9.79 and 14.04, respectively. The model generated by the random forest (RF) algorithm had the largest R and the smallest RMSE, hence, the most accurate in predicting the lead removal efficiency by the synthesized ZIF-60. The visualization of the performance of the individual models is presented in Figure 10. The findings show that machine learning algorithms can be used to obtain valuable information regarding the performance of adsorbents in the removal of contaminants from aqueous systems. The performance of the models can be enhanced provided that more dataset is used in training the models which are used for predicting the adsorbent’s performance in removing water pollutants.

### 3.7. Comparison of Adsorption Parameters with Other Studies

This study revealed the excellent removal efficiency and adsorption capacity led by ZIF-60. The results achieved are remarkable and better than those obtained using many MOF materials. Table 9 gives a comparison between the performance of ZIF-60 and other MOF materials taking note of the process parameters, kinetic models, adsorption isotherm and thermodynamic parameters.

## 4. Conclusions

ZIF-60 was synthesized in this study, and its application in the field of adsorptive removal of contaminants from water was pioneered. The crystal phase, surface functional groups and thermal stability of the synthesized ZIF-60 were investigated using XRD, FTIR and TGA characterization techniques, respectively. Using a multivariate approach (RSM), the effect of process parameters including adsorbent dose, initial concentration and temperature on the performance of ZIF-60 toward the removal of lead from aqueous solutions was studied. The experimental results revealed up to 99% lead removal when a ZIF-60 dose of 0.075 g/L and 50 ppm lead concentration were used. Further, the results of the RSM technique were used to generate regression models, and the quadratic model proved to be the most accurate with the highest R^2^. Analysis of variance was used to ensure that the quadratic model reported was valid. The results from the RSM techniques provided information regarding the selection of the process parameters that were then used for adsorption kinetics, isotherm and thermodynamics studies. The result of the adsorption kinetics exercise revealed that experimental data fitted well to both pseudo-first- and second-order kinetic equations which suggest that uptake of lead onto ZIF-60 is a complex process involving both physisorption and chemisorption. The adsorption isotherm data fitted well to the Langmuir and Redlich–Peterson isotherms indicating that the process is macroscopic homogeneous adsorption and not multi-layer adsorption on a heterogamous surface. The maximum monolayer adsorption capacity of ZIF-60 was computed using the Langmuir isotherm and was found to be 1905.0 mg/g, a value greater than what was obtained when many adsorbents were used. The thermodynamics investigation showed that the adsorption process is endothermic and spontaneous in nature indicating that an increase in temperature is of positive influence on the process. Finally, the experimental data obtained was used to test the predictive prowess of several machine learning algorithms including the ANN, SVM and RF. Based on the R and RMSE, the model generated by the random forest algorithm showed the highest efficiency in the prediction of lead removal by ZIF-60 in an aqueous system.

## Figures and Tables

**Table 1:** Independent variables and their respective levels as employed in the RSM modeling.

Factors	Level		
	−1	0	1
A = adsorbent dose (g/L)	0.025	0.05	0.075
B = lead concentration (ppm)	50	100	150
C = temperature (°C)	25	35	45

**Table 2:** Descriptive statistics of the dataset used in machine learning study.

	Adsorbent Dose (g/L)	Lead Concentration (mg/L)	Temperature (°C)	Removal Efficiency (%)
Mean	0.0394	128.462	30.769	63.310
Standard Error	0.004	22.474	1.586	6.149
Median	0.025	100	25	68.980
Mode	0.025	50	25	#N/A
Standard Deviation	0.020	114.593	8.086	31.353
Sample Variance	0.0004	13,131.540	65.385	983.030
Kurtosis	−0.727	4.284	−0.727	−1.201
Skewness	0.960	2.011	0.960	−0.428
Range	0.05	490	20	91.75
Minimum	0.025	10	25	7.74
Maximum	0.075	500	45	99.49
Sum	1.025	3340	800	1646.062
Count	26	26	26	26

**Table 3:** RSM design matrix for lead removal experiments.

Std	Run	A: Dose	B: Concentration	C: Temperature	Removal Efficiency
		g/L	Ppm	°C	%
1	1	0.025	50	25	71.5
12	2	0.05	150	35	49.6
3	3	0.025	150	25	28.3
8	4	0.075	150	45	74.1
13	5	0.05	100	25	77.4
10	6	0.075	100	35	96.9
14	7	0.05	100	45	94.8
2	8	0.075	50	25	99.5
7	9	0.025	150	45	29.7
9	10	0.025	100	35	55.4
4	11	0.075	150	25	62.3
6	12	0.075	50	45	99.0
11	13	0.05	50	35	98.4
5	14	0.025	50	45	66.5
15	15	0.05	100	35	84.2

**Table 4:** Analysis of variance result for the lead removal model.

Source	Adj SS	DF	Adj MS	F-Value	*p*-Value
Model	7911.21	9	879.02	23.05	0.0015
A-Dose	3256.58	1	3256.58	85.40	0.0002
B-Concentration	3644.66	1	3644.66	95.58	0.0002
C-Temperature	62.75	1	62.75	1.65	0.2558
AB	40.05	1	40.05	1.05	0.3524
AC	27.98	1	27.98	0.7337	0.4308
BC	43.24	1	43.24	1.13	0.3356
A^2^	194.58	1	194.58	5.10	0.0734
B^2^	301.82	1	301.82	7.92	0.0374
C^2^	3.87	1	3.87	0.1014	0.7630
Residual	190.66	5	38.13		
Cor Total	8101.86	14			

**Table 5:** Constants for lead adsorption kinetic models.

Pseudo First Order Model			Pseudo Second Order Model			Intraparticle Diffusion Model		Avrami Model			
$q_{t}=q_{e}\left(1-e^{-k_{1}t}\right)$			$q_{t}=\frac{q_{e}^{2}k_{2}t}{1+q_{e}k_{2}t}$			$q_{t}=k_{id}t^{0.5}$		$q_{t}=q_{e}\left(1-e^{\frac{-{\left(k_{a}t\right)}^{n}}{n}}\right)$			
*K*_1_ (min^− 1^)	*q*_e_ (mg/g)	R^2^	*K*_2_ (g/mg min)	*q*_e_ (mg/g)	R^2^	*K_id_* (mg g^−1^ min^− 1^)	R^2^	*q*_e (_mg/g)	*k*_a_ (min^−1^)	*n*	R^2^
0.0071	1984.5	0.9940	3.94 × 10^−6^	2243.2	0.9723	69.48	0.6828	1947.9	0.0086	1.2064	0.9980

**Table 6:** Model parameters for the adsorption isotherms under study.

Langmuir Adsorption Isotherm			Freundlich Adsorption Isotherm			Redlich–Peterson Adsorption Isotherm			
$q_{e}=\frac{q_{\max}K_{L}C_{e}}{1+K_{L}C_{e}}$			$q_{e}=K_{F}C_{e}{}^{1/n}$			$q_{e}=\frac{K_{R}C_{e}}{1+a_{R}C_{e}{}^{g}}$			
*q*_max_ (mg/g)	*K_L_* (L/mg)	R^2^	*N*	*K_F_* (L/mg)	R^2^	*K_R_* (L/g)	*A*_R_ (L/mg)	*g*	R^2^
1905.0	1.0577	0.9817	7.6	1016.3	0.9002	2794.9	1.72	0.9656	0.9866

**Table 7:** Thermodynamics parameters for lead uptake by ZIF-60.

Pollutant	T (K)	ΔG ((KJ/mol)	ΔH (KJ/mol)	ΔS (KJ/(mol·k))
Pb(II)	298	−10.47	65.34	0.25
	308	−11.95		
	318	−15.58		

**Table 8:** Hyperparameter values for the individual algorithms.

ANN		SVM		RF	
Parameter	Value	Parameter	Value	Parameter	Value
N-Fold cross validation	4	N-Fold cross validation	4	N-Fold cross validation	3
Learning rate	0.1	Kernel	Pearson VII universal kernel	Number of trees	60
Momentum	0.5	Regression optimizer	SMOreg improved	Max features	Int(log2(# predictors) + 1)
Hidden layers	1	Cost	3	Max depth	None
Training time	600	Epsilon	10–12		
		Epsilon parameter	0.001		
		Tolerance	0.01		

**Table 9:** Lead adsorption capacity comparison between ZIF-60 and other MOFs.

Material	C_o_ (ppm)	pH	Time (h)	Temp (°C)	Dosage (g/L)	*q*_max_ (mg/g)	Kinetics model	Isotherm model	Thermodynamic parameters (ΔG, ΔH (KJ/mol) ΔS(KJ/(mol·k))	Reference
ZIF-60	10–400	4	24	25	0.025	1905.0	PFO and Avrami	Langmuir and Redlich–Peterson	ΔG = −10.47, ΔH = 65.33, ΔS = 0.25	This study
MOF-5	20–100	5	6	45	0.25	658.5	PSO	Langmuir and BET	–	[60]
ZIF-8	200	5.1	2	25	0.1–2.0	1119.8	PSO	–	–	[26]
ZIF-67	200	5.1	2	25	0.1–2.0	1348.4	PSO	–	–	[26]
PAN/chitosan/UiO-66-NH_2_ MOF	−0–1000	6	1	25	0.2	441.2	PSO	Redlich–Peterson	ΔG = −8.01, ΔH = −46.78, ΔS = 0.15	[30]
Fe-MIL-101	9.12–90.12	6	3.5	30	0.5	86.2	PSO	Langmuir and Freundlich	ΔG = −2.55, ΔH = 45.35, ΔS = 0.16	[61]
MIL-101	9.12–90.12	6	3.5	30	0.5	58.0	PSO	Langmuir	ΔG = −1.38, ΔH = 18.55, ΔS = 0.07	[61]
ZIF-8@CA	5–1000	5	2	25		1321.2	PSO	Langmuir	–	[62]
nFE@ZIF-8	50	5	1	45	0.025	227.3	PSO	Langmuir	ΔG = −7.40, ΔH = 11.64, ΔS = 0.06	[63]
ZIF@GO		5	1.7	25	0.15	356.0	PSO	Langmuir	ΔG = −10.82, ΔH = 4.11, ΔS = 0.05	[64]
ZIF@NiTiO_3_	100	5	2	25	0.67	155.0	PSO	Langmuir	–	[65]
carbon sphere@ZIF-8	100–600	5.5	5	25	1	310.5	PSO	Langmuir	–	[66]
ZIF-8	20	6.5	2	25	1	1782.3	PSO	Langmuir	–	[25]
ZIF-67	20	6.5	2	25	1	1972.1	PSO	Langmuir	–	[25]
ZIF-8@GO-COOH	20–500	6	1	25	1	410.1	PFO and PSO	Langmuir	ΔG = −22.96, ΔH = 33.23, ΔS = 0.18	[29]

## Data Availability

Data are contained within the article. For any additional data request, the authors may be contacted.
